# Cloning, expression and characterization of metalloproteinase HypZn from *Aspergillus niger*

**DOI:** 10.1371/journal.pone.0259809

**Published:** 2021-11-11

**Authors:** Peng Song, Wei Xu, Kuiming Wang, Yang Zhang, Fei Wang, Xiuling Zhou, Haiying Shi, Wei Feng

**Affiliations:** School of Life Sciences, Liaocheng University, Liaocheng, China; Russian Academy of Medical Sciences, RUSSIAN FEDERATION

## Abstract

A predicted metalloproteinase gene, *HypZn*, was cloned from *Aspergillus niger* CGMCC 3.7193 and expressed in *Pichia pastoris* GS115, and the physicochemical characteristics of recombinant HypZn were investigated after separation and purification. The results showed that the specific activity of the purified HypZn reached 1859.2 U/mg, and the optimum temperature and pH value of HypZn were 35°C and 7.0, respectively. HypZn remained stable both at 40°C and at pH values between 5.0 and 8.0. The preferred substrate of HypZn was soybean protein isolates, and the *K*_m_ and *V*_max_ values were 21.5 μmol/mL and 4926.6 μmol/(mL∙min), respectively. HypZn was activated by Co^2+^ and Zn^2+^ and inhibited by Cu^2+^ and Fe^2+^. The degree of soybean protein isolate hydrolysis reached 14.7%, and the hydrolysates were of uniform molecular weight. HypZn could tolerate 5000 mM NaCl and completely lost its activity after 30 min at 50°C. The enzymological characterizations indicated that HypZn has great application potential in the food industry, especially in fermented food processing.

## 1 Introduction

Metalloproteinases are proteolytic enzymes that contain Zn^2+^, Co^2+^, Ni^2+^ and other metal ions in their active centers, and they rely on these metal ions to catalyze the hydrolysis of peptide bonds. Metal ions are responsible for activating water molecules during the catalytic process, and activated water molecules nucleophilically attack the carbonyl groups of peptide bonds, thus facilitating the hydrolysis of substrates [1]. Metalloproteinases differ greatly in their primary structure, but their active sites are relatively conserved [2]. Microbial metalloproteinases have high proteolytic ability and show good application prospects in washing, printing and dyeing, feed and especially food. In particular, some low/high temperature-resistant enzymes can save industrial energy, reduce industrial pollution and provide an important source of green additives [3].

Microbial metalloproteinases have been studied in the past and are mainly derived from bacteria. Some examples of such bacteria include *Pseudomonas* [4–6], *Vibrio* [7–9], *Proteus* [10], *Erwinia* [11], *Bacillus* [12,13], *Escherichia* [14], *Alteromonas* [15] and *Serratia* [16]. There are relatively few reports on metalloproteinases from fungi [17–20], partly because most of the metalloproteinases produced by fungi are intracellular enzymes, which are difficult to detect and isolate and are prone to losing activity during the process of wall breaking. However, it is necessary to explore proteases from fungi to discover valuable new enzymes and study the catalytic mechanism of metalloproteinases.

Extracellular expression by heterologous expression systems is a common method for studying fungal metalloproteinases. The genome of *Aspergillus niger* CBS 513.88 was analyzed [21], and a new suspected metalloproteinase (Gene ID:4986996, named *HypZn*) was identified. After its cloning and expression in *Pichia pastoris* GS115, the enzymatic properties of recombinant HypZn and protein substrate hydrolysis were systematically studied, and the potential application of HypZn in the food field was especially discussed.

## 2 Materials and methods

### 2.1 Materials

#### 2.1.1 Strains and plasmids

The shuttle plasmid pPIC9K (Invitrogen) was used for gene cloning and expression. *E*. *coli* JM109 (stored in our laboratory) was used for plasmid amplification and bacterial transformation; *P*. *pastoris* GS115 (Invitrogen) was the host bacteria for gene expression. *A*. *niger* CGMCC 3.7193 was stored in the China General Microbial Culture Collection Management Center (CGMCC) and used to clone the HypZn gene.

#### 2.1.2 Media

Potato dextrose agar (PDA) medium contained 2% potato and 2% glucose (a solid medium with 1% agar powder). Luria-Bertani (LB) medium (10% tryptone, 5% yeast extract and 10% NaCl), yeast extract peptone dextrose (YPD) medium (1% yeast extract, 2% peptone and 2% dextrose), minimal dextrose (MD) medium (1.34% yeast nitrogen base without amino acids, 2% dextrose and 0.00004% biotin), buffered glycerol-complex (BMGY) medium (1% yeast extract, 2% peptone, 1.34% yeast nitrogen base without amino acids, 100 mM potassium phosphate at pH 6.0, and 1% glycerol) and buffered methanol-complex (BMMY) medium (1% yeast extract, 2% peptone, 1.34% yeast nitrogen base without amino acids, 100 mM potassium phosphate at pH 6.0, and 0.5% methol) were prepared with the recommended components and according to the methods in the *Pichia pastoris* expression manual (Version F, Invitrogen).

#### 2.1.3 Restriction enzymes and reagents

The restrictive endonucleases *Sna*BΙ and *Xba*Ι, Phusion high fidelity DNA polymerase and T_4_ DNA ligase were purchased from Thermo Fisher Scientific. Plasmid extraction kits, DNA purification and recovery kits, and BCA protein concentration determination kits were purchased from Beijing Solarbio Science & Technology Co., Ltd. The RNA extraction kit and the first-strand cDNA reverse transcription kit were purchased from Takara Co., Ltd. (Dalian).

Casein was purchased from Sigma, and soybean protein isolates were purchased from Beijing OKA Biotechnology Co., Ltd. Trypsin 1:2500 (from pig pancreas), pepsin 1:3000 (from pig stomach) and papain 1:2000 were purchased from Sangon Bioengineering (Shanghai) Co., Ltd., and the *Aspergillus* acid protease 1:50000 and alkaline protease 1:200000 were purchased from Beijing Coolaber Technology Co., Ltd. HPLC protein molecular weight standards (cytochrome, 12500 Da; aprotinin 6500 Da, bacitracin 1450 Da, Gly-Gly-Tyr-Arg, 451 Da and Gly-Gly-Gly, 189 Da) were purchased from the Chinese Institute of Metrology.

### 2.2 Methods

#### 2.2.1 Cloning of the HypZn gene

The spores of *A*. *niger* CGMCC were stored at -80°C, inoculated onto PDA medium and cultured at 32°C and 240 rpm for 24 h. Then, the mycelia were collected. Total RNA was extracted from *A*. *niger* and identified according to the kit instructions, and first-strand cDNA was synthesized. The forward and reverse primers were designed as follows: 5’- CCTGACATCCGGGCCC-3’ and 5’-TGCTCTAGATCCGTACATCTTTAATTGCCA-3’. These primers were used for gene amplification from the synthesized cDNA. PCR conditions were as follows: 95°C for 5 min, denaturation at 95°C for 10 s, annealing at 60°C for 30 s, and extension at 72°C for 1.5 min for 30 cycles, followed by extension at 72°C for 10 min. The PCR product was recovered by a DNA purification kit, ligated into the pPIC9K plasmid, and then transformed into *E*. *coli* JM109. Positive clones were screened and sequenced to confirm the correct sequence. The recombinant plasmid was named pPIC9K- HypZn.

#### 2.2.2 Construction and induction of expression of recombinant *Pichia pastoris* GS115

The recombinant plasmid pPIC9K-HypZn was extracted, and after *Sac* I linearization, it was electrically transformed into *P*. *pastoris* GS115. on solid MD plates, His^+^ recombinants were identified, and then the clones were transferred to YPD plates with 0.5, 1.0 and 2.0 mg/mL genetic mycin (G418) to select colonies with high copy number expression. A single resistant colony was selected from a 2 mg/mL G418 plate and inoculated in 25 mL BMGY medium. The culture was oscillated at 30°C and 250 rpm until the OD_600_ value reached 4.0. BMMY medium (50 mL) was inoculated at 1% (v/v) and cultured under the same conditions. Methanol (0.5% (v/v)) was added every 24 h for expression induction. After 120 h, the supernatant was collected by centrifugation. Enzyme activity was detected, and protein expression was analyzed by SDS-PAGE (12% separation gel).

#### 2.2.3 Purification and determination of recombinant protease enzyme activity

The supernatant was centrifuged at 4°C for 20 min at 16000×*g* and then slowly added to the saturated ammonium sulfate solution until the concentration of ammonium sulfate was 85%. The precipitates were collected by centrifugation under the same conditions. The precipitates were suspended in 20 mM pH 7.0 Tris-HCl buffer (20 mM Tris solution was prepared, and adjust the pH value was adjusted to pH 7.0 with HCl) and concentrated with PEG 8000. The concentrated supernatant was loaded into a Sefacryl S-200 column balanced with 20 mM Tris-HCl buffer (pH 7.0). The proteins were eluted in one column volume of Tris-HCl buffer at a flow rate of 1.0 mL/min. The HypZn components were collected and concentrated with an ultrafiltration membrane.

According to the China Standard QB/T1803 General Experimental Methods for Industrial Enzyme Preparation [22], casein was used as a substrate to detect HypZn activity: briefly, 1 mL of the diluted enzyme sample was mixed with equal volumes of 1% (w/v) casein in 50 mM phosphate-citrate buffer (pH 7.0; 50 mM citric acid solution was prepared, and its pH value was adjusted to 7.0 with sodium phosphate), followed by incubation at 35°C for 10 min. The reaction was terminated with 2 mL of 10% (w/v) trichloroacetic acid (TCA), and the mixture was centrifuged at 14,000g for 10 min to remove protein precipitate from the tyrosine released by the protease. The absorbance of the supernatant was measured at 280 nm to determine the amount of tyrosine released during the reaction. The standard curve was created using tyrosine ranging from 0 to 50 mg/mL. One unit of enzymatic activity was defined as the amount of enzyme needed to catalyze the release of 1 μg of tyrosine per min at 35°C and pH 7.0. The protein concentration was determined using the BCA Protein Concentration Assay Kit.

#### 2.2.4 Influence of pH value and temperature on HypZn activity and stability

HypZn activity was measured in different buffer liquid systems (pH 3.0–10.0) to determine the effect of pH on HypZn activity. The buffers described above were used to dissolve HypZn and the mixture was kept at room temperature (25°C) for 180 min. Then, the enzyme activity was detected under optimal pH conditions, and the effect of pH on the stability of the enzyme was investigated. The buffer solution used was as follows: phosphate-citrate buffer (50 mM, pH 3.0–8.0; 50 mM citric acid solution was prepared, and the pH value was adjusted to 3.0–8.0 with sodium phosphate); phosphate buffer (50 mM, pH 8.0–9.0; 0.2 M disodium hydrogen phosphate solution and 0.2 M sodium dihydrogen phosphate solution were prepared, the two solutions were mixed in different proportions to adjust the pH to 8.0–9.0, and the mixed solutions were diluted to 50 mM) and Borax-sodium hydroxide buffer (50 mM, pH 9.0–10.0; a 50 mM Borax solution was prepared, and the pH value was adjusted to 9.0–10.0 with sodium hydroxide).

The activity of HypZn was detected at 20°C to 50°C, and the effect of temperature on HypZn activity was investigated. HypZn was dissolved in the most stable pH buffer and incubated at 20°C to 50°C for 30 to 180 min to investigate the effect of temperature on the stability of the recombinant enzyme.

#### 2.2.5 Effects of metal ions and inhibitors on HypZn activity

Different metal ions or inhibitors were added to the enzyme activity detection system, and the effects of various metal ions and inhibitors on the enzyme activity of HypZn were investigated. The control was prepared with none of these molecules added.

#### 2.2.6 Dynamic parameter analysis

Using casein or soybean protein isolates as substrates, kinetic analysis of the enzyme reaction was carried out under the optimal reaction conditions. The kinetic parameters *K*_m_ and *V*_max_ were calculated by the Lineweaver-Burk plotting method [23]. *K*_cat_ = *V*_max_/[E], where [E] is the enzyme concentration.

#### 2.2.7 HPLC analysis of hydrolyzed soybean protein isolates and their hydrolyzed products

Using 1% soybean protein isolates as a substrate, the ability of HypZn to hydrolyze natural protein was studied: 10 U recombinant HypZn was added to a 10-mL reaction system, 0.1 g soybean protein isolates was hydrolyzed for 3 h under the optimal conditions, and the degree of hydrolysis was determined by the OPA method [24]. Trypsin, pepsin, papain, acid protease and alkaline protease were used as controls.

The molecular weight and distribution of the hydrolysates were analyzed on a TSKGEL G2000 SWXL column. The chromatographic conditions were as follows: mobile phase, 0.05 M phosphate buffer (pH 7.0) was added to a 0.3 M NaCl; column temperature, 30°C; flow rate, 0.5 mL/min; DAD detector, UV 220 nm; and injection volume, 10 μl. HPLC analysis was performed on an Agilent high-performance liquid chromatograph.

#### 2.2.8 Bioinformatics analysis

The online tool ProtParam was used to calculate the theoretical molecular weight of HypZn (http://us.expasy.org/tools/protparam.html); the online tool CDART was used to analyze the conserved structural domain in the protein amino acid sequence (https://www.ncbi.nlm.nih.gov/Structure/lexington/lexington.cgi); SignalP 5.0 was used to predict the signal peptide protein sequence (http://www.cbs.dtu.dk/services/SignalP); the HypZn family category was determined with the Merops peptidase database (http://merops.sanger.ac.uk); the amino acid sequence and the protein sequence alignment analysis for HypZn were determined using the NCBI protein blast program (http://www.ncbi.nlm.nih.gov); and PredictProtein (https://www.predictprotein.org) online was used to analyze disulfide bonds in the protein structure.

#### 2.2.9 Data analysis

The data presented are the mean values of triplicates from three separate experiments. Data were analyzed using one-way analysis of variance (ANOVA) on Statistical Package for the Social Sciences (SPSS, version 20), and the means were separated using Duncan’s multiple range test (DMRT) at a P<0.05 significance level.

## 3 Results and discussion

### 3.1 Cloning and characterization of the HypZn gene

**Fig 1:**
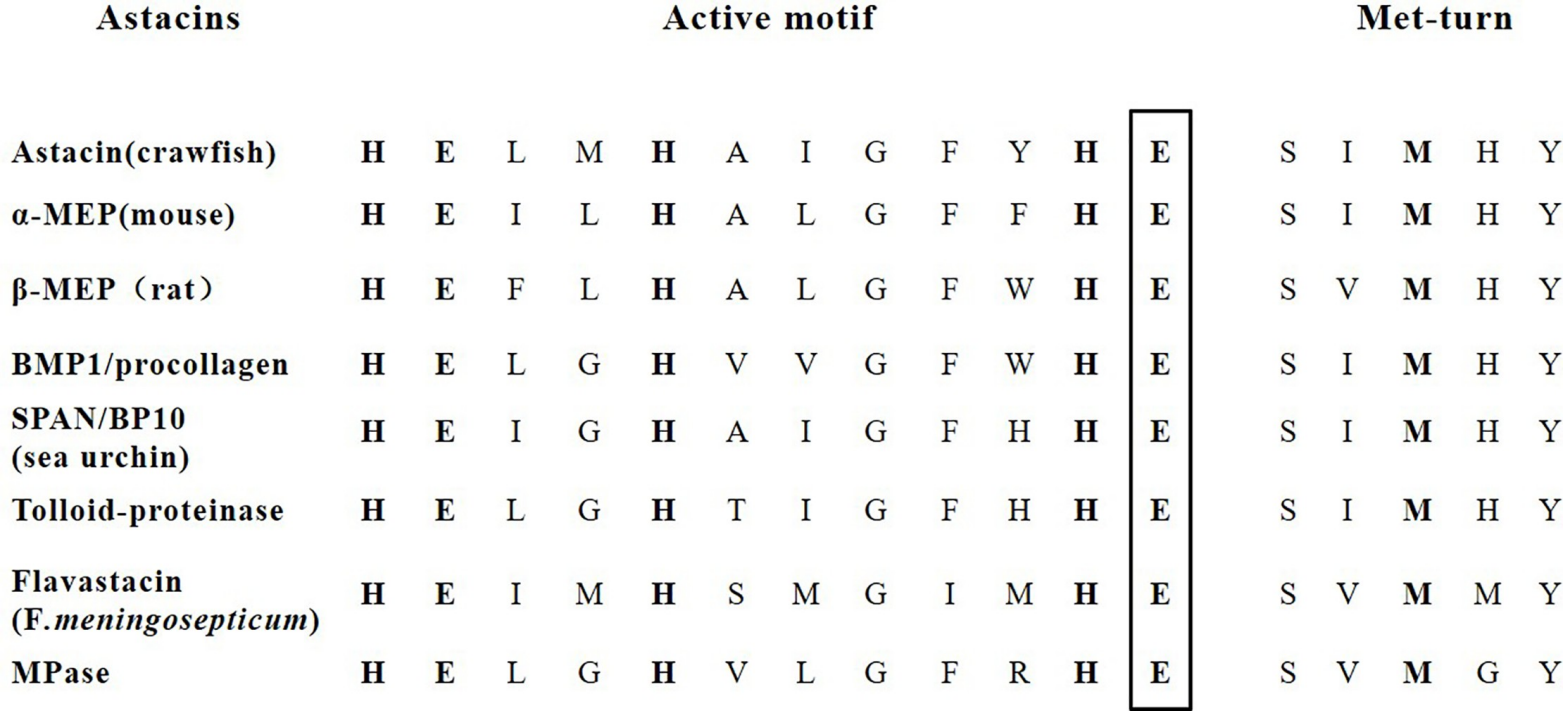
Marker conservative sequences of astacin-like metalloendopeptidases: The extended motif of the active site and Met turn.

The complete coding sequence (CDS) of HypZn was amplified by RT-PCR. The full-length HypZn gene contained 858 bp, and it encoded 285 amino acids, with a theoretical molecular weight of 31.6 kDa. No signal peptide sequence was found in the HypZn protein sequence analysis. The conserved domain retrieval tool (CDART) of the NCBI database showed that this enzyme belongs to the zinc-dependent metalloprotease (Metzincin) superfamily; it has a conserved HEXXHXXGXXH/D motif (X represents any amino acid) and a "met-turn" structure in the C-terminus (Fig 1) [25].

Metzincin consists of two main branches: an astacin-like protease and adamalysin/reprolysin-like protease [26]. Further analysis showed that HypZn is an astacin-like protease because the only astacin-like proteases are glutamate (E) residues that follow the conserved third histidine (H) motif, and HypZn fits this typical structure (Fig 1) [27]. To the best of our knowledge, HypZn is the first report of an astacins-like protease in fungi. Astacin was isolated in 1967 from the alimentary track of crayfish *Astacus astacus* L. These enzymes were mainly found in representatives of the animal kingdom except one found in gram-negative bacteria *Flavobacterium meningosepticum* [27].

### 3.2 Construction of recombinant bacteria and gene expression

**Fig 2:**
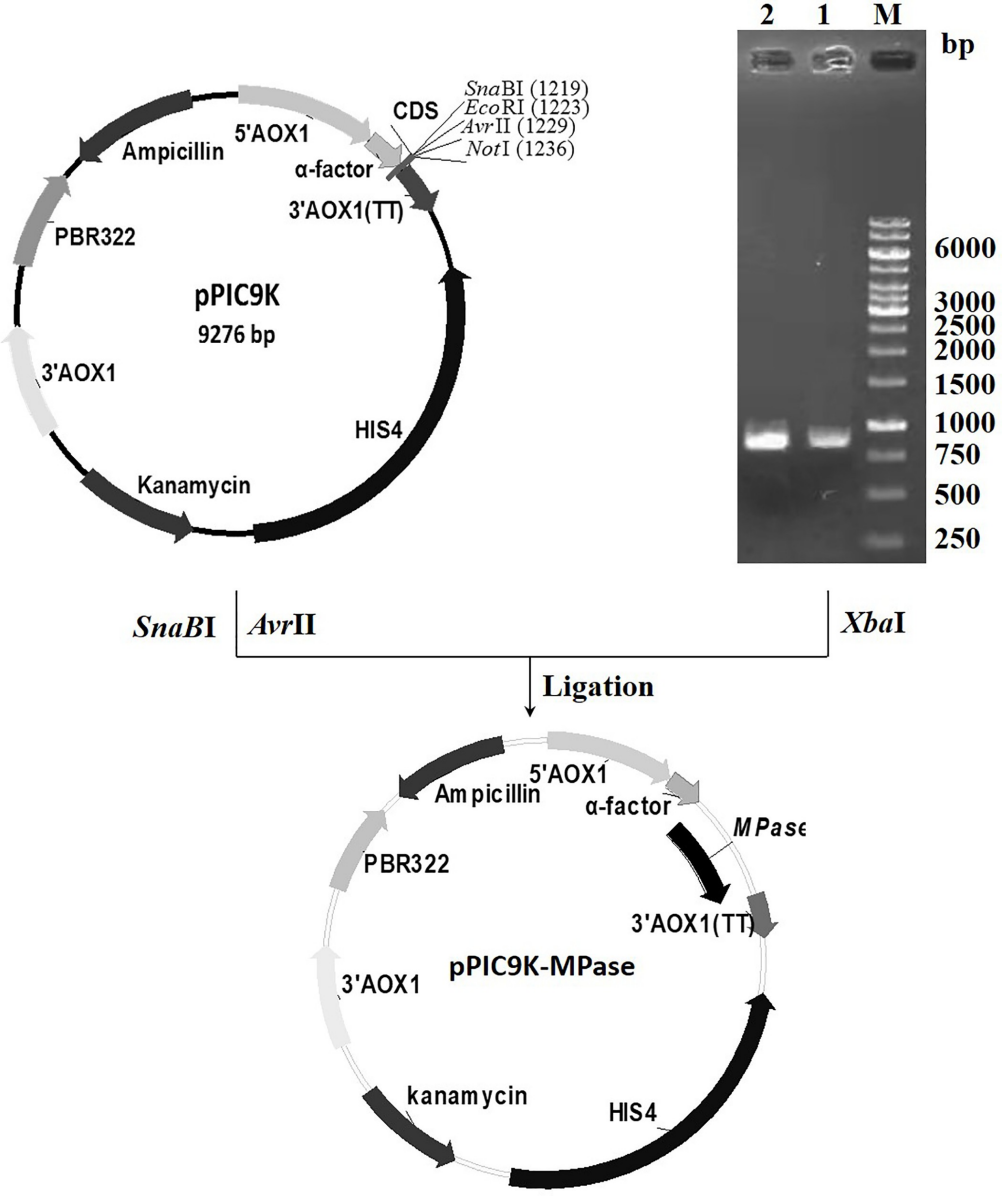
Construction of the expression vector pPIC9K-HypZn. The five bands from top to bottom are 4125 bp, 2555 bp, 1827 bp, 1241 bp and 377 bp.

**Fig 3:**
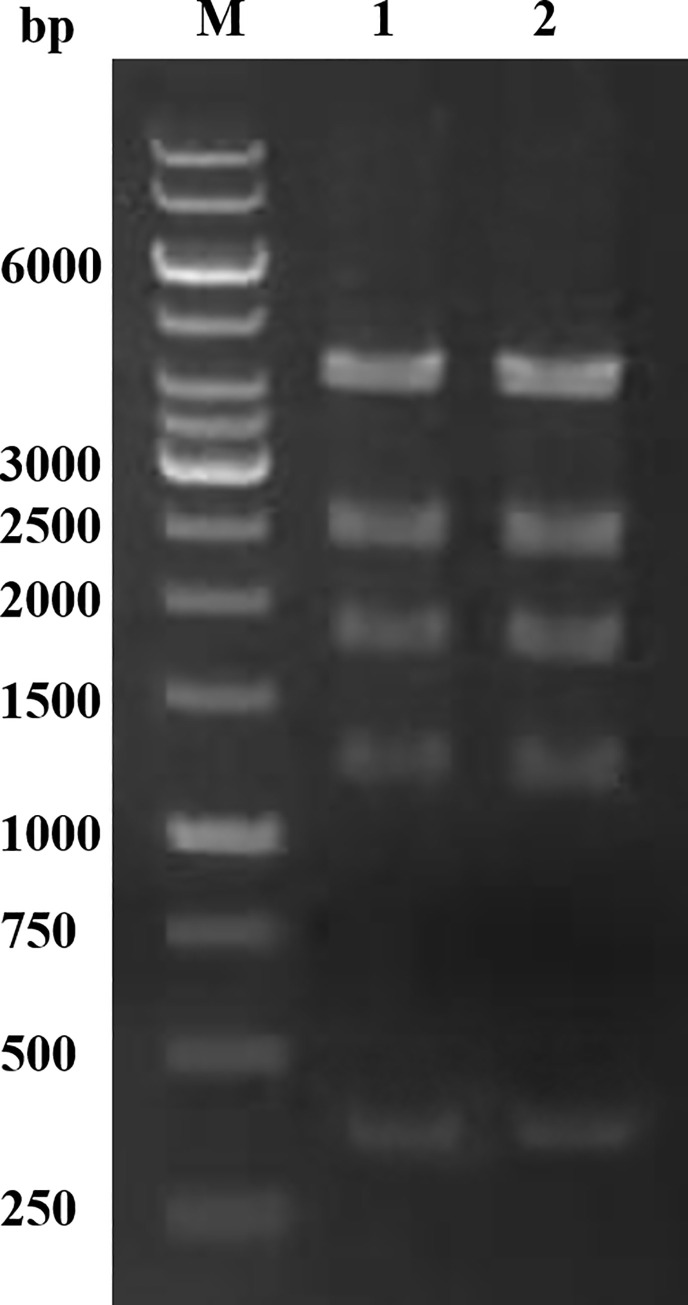
Characterization of the expression vector pPIC9K-HypZn with *Pst* Ι.

The HypZn gene fragment was amplified by PCR and then digested by *Xba* Ι. This fragment was ligated in the *SnaB*Ι/*AvrII*-digested PPIC9K vector, and the expression vector PPIC9K-HypZn was successfully constructed (Fig 2). *E*. *coli* JM109 was transformed with pPIC9K-HypZn, and the recombinant plasmid was extracted after amplification; the sequence was verified by *Pst* Ι digestion. The agarose gel clearly showed five bands of 4125 bp, 2555 bp, 1827 bp, 1241 bp and 377 bp (Fig 3), which were consistent with the theoretical values. Sequencing also confirmed that the sequence was correct. The confirmed recombinant plasmid was linearized by *Sal* Ι and electrically transformed into *P*. *pastoris* GS115, and recombinant *P*. *pastoris* GS115-HypZn expressing a high copy number was identified. *P*. *pastoris* GS115, transformed into the pPIC9K empty plasmid, was used as the control. After gene expression induction in BMMY medium with methanol, the supernatants of the recombinant strain and the control were collected to detect HypZn protease activity. Under the same conditions, the specific activity of the supernatant of the recombinant strain was 1223.4 U/mg, while that of the control strain was 1.2 U/mg.

### 3.3 Purification of the recombinant proteases

**Fig 4:**
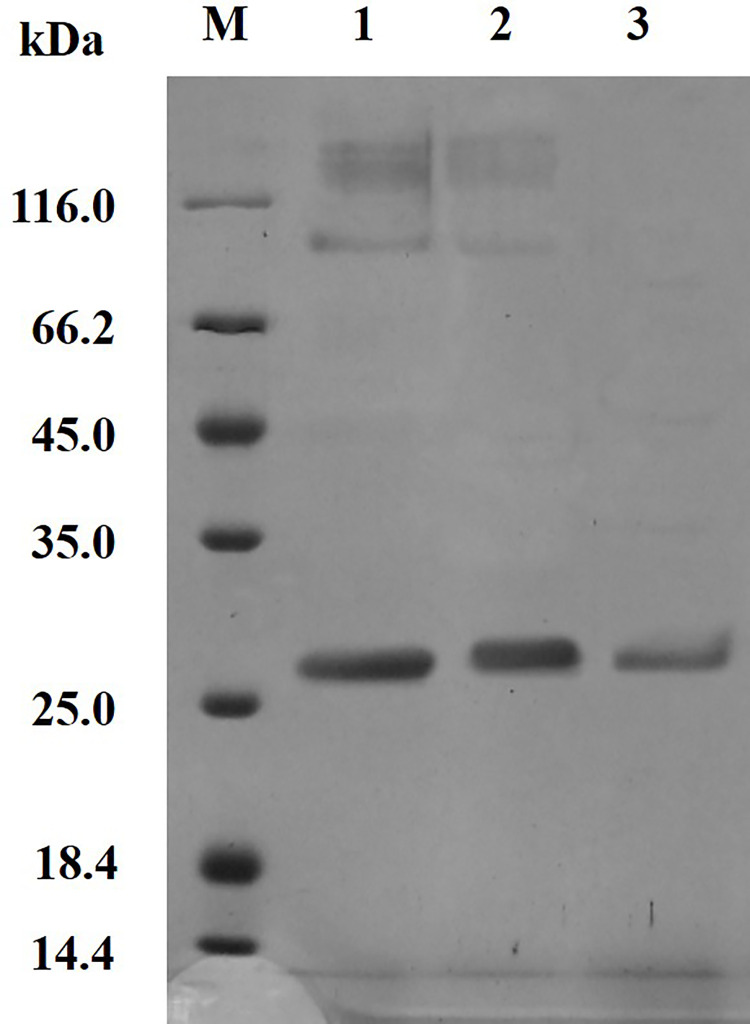
SDS-PAGE analysis of the purification of recombinant protease HypZn. M: Protein molecular weight standard; 1: Crude enzyme solution; 2: Enzyme liquid precipitated by ammonium sulfate; 3: Enzyme solution purified by chromatographic column chromatography.

The expression of the target proteins was analyzed by SDS-PAGE. The protein bands ranged from 25.0 to 35.0 kDa (Fig 4), which was consistent with the theoretical molecular weight. The supernatant was precipitated by ammonium sulfate and separated by gel chromatography, and HypZn presented a clear single band in the SDS-PAGE results (Fig 4). The final recovery rate was 52.9%, and the purification fold was 1.5. After purification, the specific activity of HypZn reached 1859.2 U/mg (Table 1).

**Table 1:** Purification of recombinant HypZn.

Purification steps	Total protein (mg)	Total activity (U)	Specific activity (U/mg)	Purification fold	Recovery rate (%)
Crude extract	80.0	97868.3	1223.4	1	100
85% Ammonium sulfate precipitation	64.2	89857.9	1399.7	1.1	80.0
Sephacryl S-200 chromatography	42.3	78644.5	1859.2	1.5	52.9

As an efficient heterologous expression system, *P*. *pastoris* GS115 expresses foreign proteins at high levels. With the α-factor of PPIC9K, foreign proteins can be directly secreted into the fermentation broth, and the level of protein secreted by the *P*. *pastoris* expression system is low. This makes purifying the target protein relatively easy [28]. The HypZn supernatant was precipitated by ammonium sulfate, dialysis and Sephacryls-200 column chromatography to obtain the purified protein. Subsequently, we examined the purified protein band by SDS-PAGE: compared with astacin from *Astacus astacus L*., which consists of 200 amino acid residues with a molecular mass of 20 kDa [29], HypZn is much larger with a molecular mass of more than 25 kDa and consists of 285 amino acid residues. The results indicate that there are significant structural differences between animal-sourced astacin and fungus-sourced astacin.

### 3.4 Activity and stability of HypZn under different pH and temperature conditions

**Fig 5:**
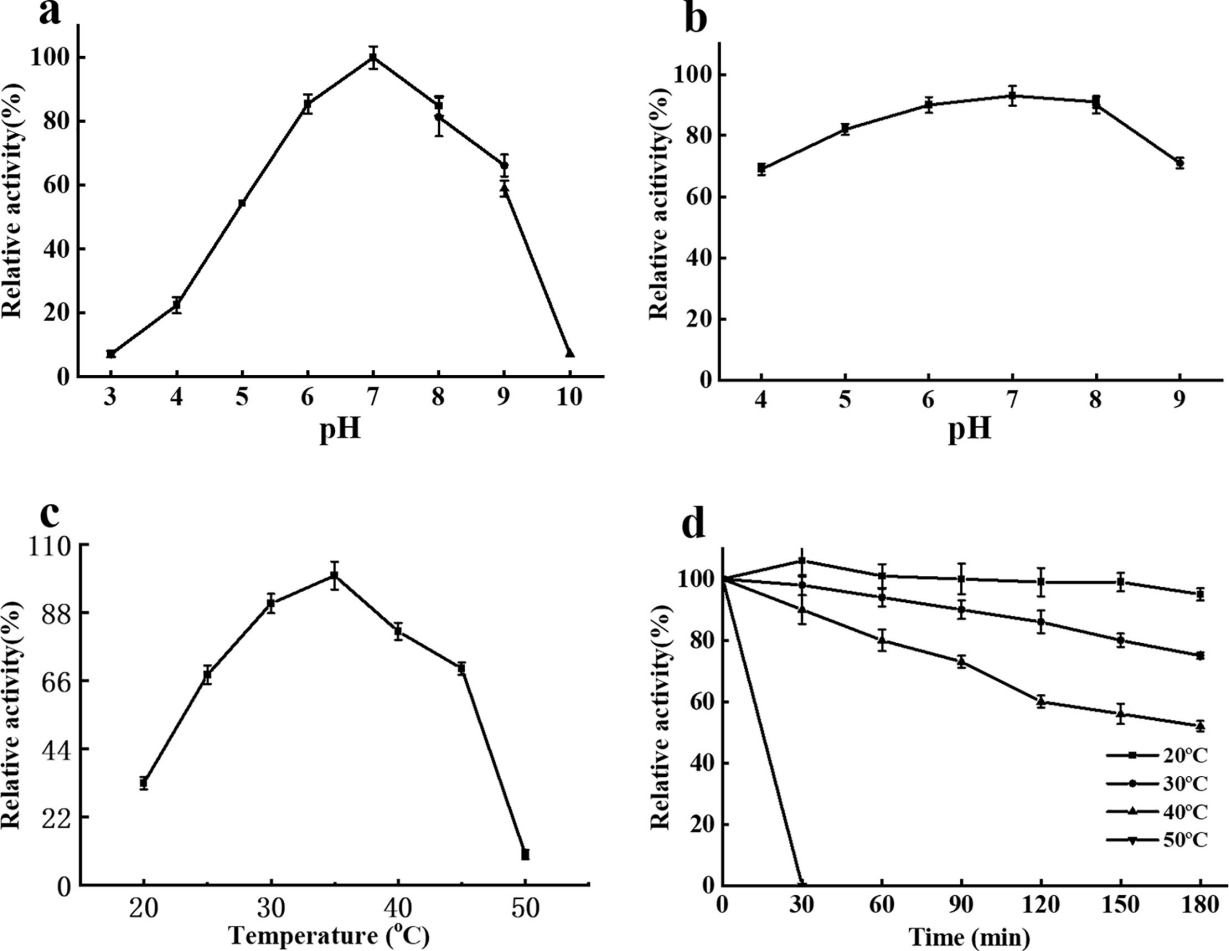
Enzyme activity and stability profiles of HypZn at various pH values and temperatures using soy protein isolates as substrates. A: The effect of pH on the activity of HypZn; B: The effect of pH on the stability of HypZn; C: The effect of temperature on the activity of HypZn; D: The effect of temperature on the stability of HypZN. The data are presented as the mean ± standard deviation of the three measurements.

The optimal pH for HypZn activity was 7.0. HypZn activity increased rapidly from pH 4.0–7.0. The activity decreased rapidly from pH 9.0–10.0. At pH 6.0–8.0, more than 80% of the enzyme activity was maintained (Fig 5A). HypZn was most stable at pH 7.0. When maintained at pH 5.0–8.0 at 25°C for 180 min, HypZn maintained more than 80% of its enzymatic activity (Fig 5B).

The optimal reaction temperature for HypZn activity was 35°C. The activity of HypZn increased significantly at 20–35°C and decreased significantly at 45–50°C (Fig 5C). The activity of HypZn remained above 80% after incubation at 20°C or 30°C for 180 min. When the incubation temperature was increased to 40°C, the enzyme activity decreased significantly (< 60%). When the incubation temperature was increased to 50°C, the enzyme activity was completely lost after 30 min (Fig 5D).

Compared with the reported metalloproteinases [30,31], the optimal reaction temperature of HypZn is lower (35°C), and it is more easily inactivated at high temperature (it is completely inactivated after a 30-min incubation at 50°C), which means that HypZn can hydrolyze protein substrates under the milder conditions used in food processing, and it can be inactivated at a lower temperature after the reaction is completed. Thus, the flavor of food is not affected [1]. Generally, astacin is stable in neutral and weakly acidic media, but at pH < 4.0, the protein is irreversibly inactivated [27]. However, HypZn retained its activity and maintained more than 60% activity after incubation for 180 min at pH 4.0, which means that HypZn can be applied in industry.

Analysis of the HypZn amino acid sequence shows that it contains only two cysteine residues, and PredictProtein online analysis (https://www.predictprotein.org) shows that the cause of the poor thermal stability of HypZn may be the protein structure, which lacks disulfide bonds. The disulfide bond between or within protein subunits was considered to be an important factor contributing to thermal stability [32].

### 3.5 Effects of different metal ions and inhibitors on HypZn

Activating HypZn with Zn^2+^ and Co^2+^ significantly increased the activity of HypZn (188.7%). In in vitro catalysis, Co^2+^ should be used as the HypZn cofactor. Cu^2+^ and Fe^2+^ had a strong inhibitory effect on HypZn (Table 2). The metal-chelating agents EDTA and EGTA strongly inhibited HypZn activity, indicating that HypZn catalysis requires metal cofactors, indicating that HypZn is a metalloproteinase [33]. *β*-Mercaptoethanol and DTT had almost no effect on enzyme activity, indicating that the HypZn structure does not contain a disulfide bond [34]. The cysteine protease inhibitor E-64 had little effect on enzyme activity, indicating that the HypZn active site does not contain cysteine and that it is not a mercaptoprotease [35]. The serine protease inhibitor PMSF did not affect enzyme activity, indicating that this enzyme is not a serine protease [36]. The aspartate protease inhibitor pepstatin had no effect on enzyme activity, indicating that the enzyme is not an aspartate protease (Table 2) [37]. HypZn can normally hydrolyze substrates in the presence of 5000 mM NaCl, which is beneficial for its use in many fermented foods, such as fermented sausage and cheese production and soy sauce fermentation, where high levels of salt are commonly added.

**Table 2:** Effect of metal cations and protease inhibitors on the activity of HypZn.

Metal ions and inhibitors	Concentration (mM)	Relative enzyme activity (%)
Co^2+^	5	202.3^b^
Zn^2+^	5	118.3^b^
Ca^2+^	5	101.3^a^
Mg^2+^	5	98.3^a^
Mn^2+^	5	97.2^a^
Cu^2+^	5	72.1^b^
Fe^2+^	5	65.2^b^
NaCl	5000	103.1^a^
EDTA	1	7.1^b^
EGTA	1	10.7^b^
β-Mercaptoethanol	1	97.1^a^
DTT	1	97.2^a^
E-64	0.05	92.3^a^
PMSF	1	100.9^a^
pepstatin	0.05	97.4^a^
control	0	100^a^

The enzyme activity of HypZn in phosphate-citrate buffer pH 7.0 at 35°C (optimal conditions) was designated as standard enzyme activity 100%. Values are reported as the mean of triplicate measurements. Different lowercase superscripts indicate statistically significant differences (p < 0.05). The SAS statistical analysis software package was used for analysis of variance.

EDTA and EGTA were incubated with the enzyme for 6 h.

### 3.6 Dynamic parameter analysis

The kinetic parameters of HypZn were determined using 0.5–50 mg/mL casein and soybean protein isolates as substrates, because both are the most representative animal protein and vegetable protein worldwide. The results are shown in Table 3. HypZn had a higher affinity for the soybean protein isolates (*K*_m_), and the efficiency of the hydrolysis of soybean protein isolates was also higher (*k*_cat_/*K*_m_). Compared with casein, soybean protein isolates are the better substrate for HypZn.

**Table 3:** Kinetic parameters of recombinant HypZn for casein and soy protein isolates.

	*K*_m_ (μmol/mL)	*V*_max_ [μmol/(mL∙Min)]	*k*_cat_ (Min^-1^)	*k*_cat_*/K*_m_ [mL/(μmol∙Min)]
Soy protein isolate	21.5	4926.6	139321.3	6480
Casein	45.3	753.3	77705.7	1715.4

### 3.7 Analysis of the results of hydrolyzing soybean protein isolates

**Fig 6:**
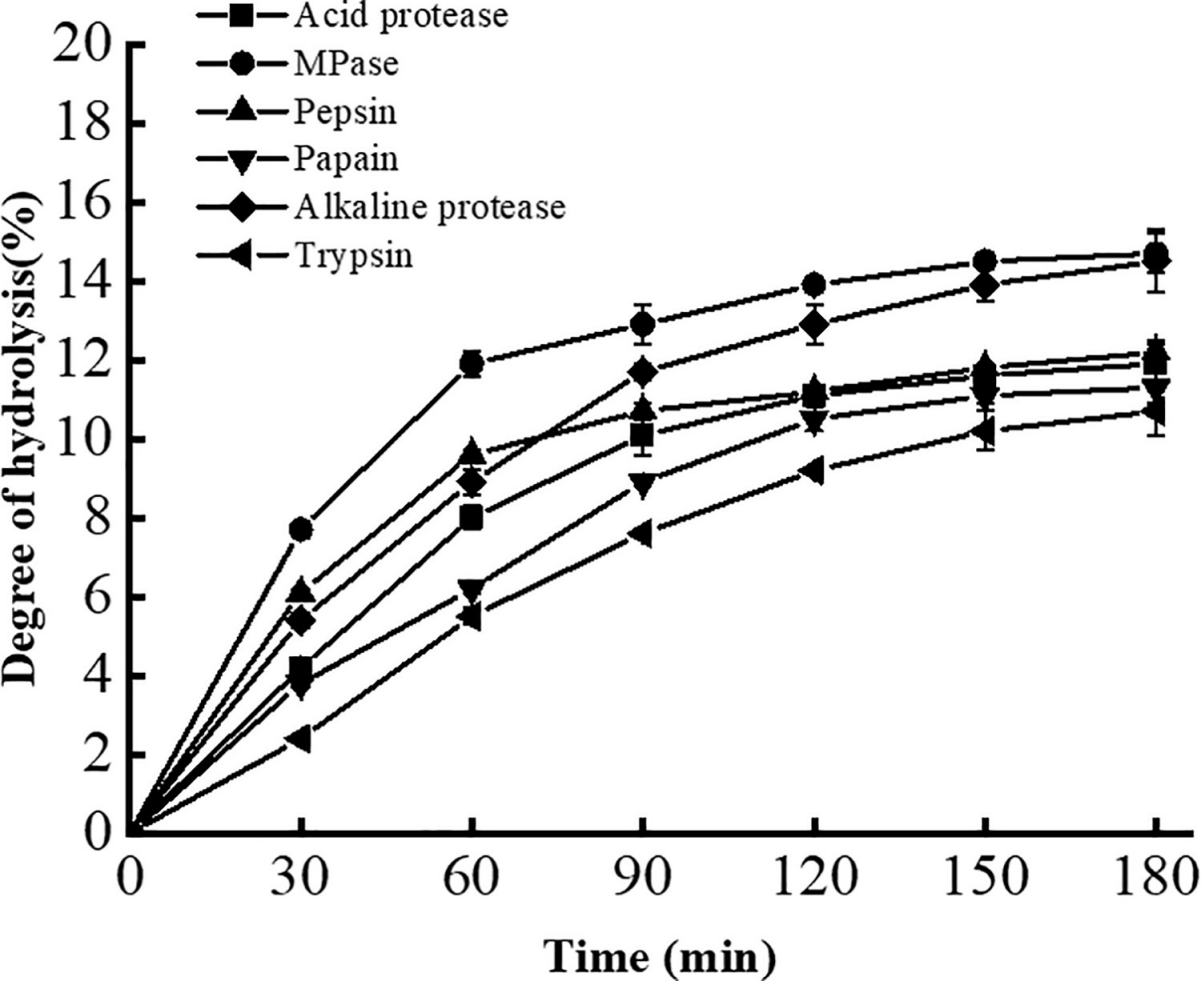
Determination of the degree of hydrolysis of soybean protein isolates by HypZn or five commonly used commercial proteases.

The degree of hydrolysis (DH, %) is the percentage of total peptide bonds that are hydrolyzed, and it is an important index of the ability of proteases to hydrolyze protein substrates [38]. Compared with five common commercial proteases, HypZn hydrolyzed soybean protein isolates with the highest degree of hydrolysis (14.7%), which is similar to that of alkaline protease (14.5%) (Fig 6). However, HypZn hydrolyzed soybean protein isolates with a higher efficiency than alkaline protease, and the maximum degree of hydrolysis of soybean protein isolates was basically completed after 60 min of reaction. In industrial applications, shortening the enzymatic hydrolysis time can prevent the breeding of miscellaneous bacteria and reduce the reaction cost.

**Fig 7:**
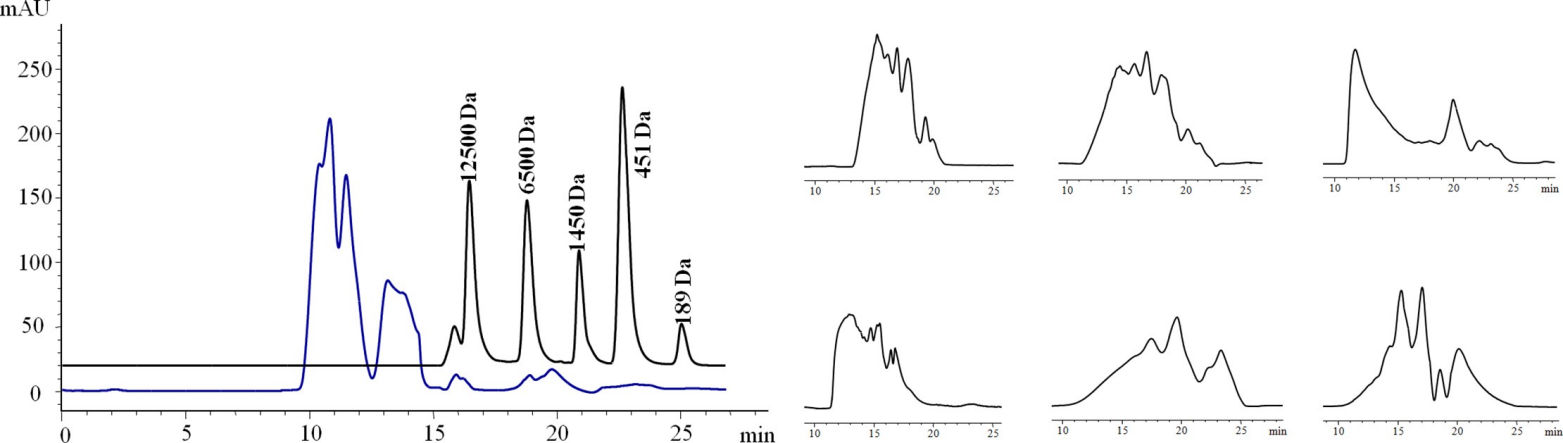
Peptide profile of soybean protein isolates hydrolyzed by HypZn or five commonly used commercial proteases. A: Peptide profile of soybean protein isolate substrate (unhydrolyzed); B: Soybean protein isolate hydrolysates treated with HypZn; C: Soybean protein isolate hydrolysates treated with trypsin; D: Soybean protein isolate hydrolysates treated with papain; E: Soybean protein isolate hydrolysates treated with acid protease; F: Soybean protein isolate hydrolysates treated with pepsin; G: Soybean protein isolate hydrolysates treated with alkaline protease.

The molecular weights of the main body (96%) of the soybean protein isolates used in this study were greater than 12,500 Da (Fig 7A). After hydrolysis by HypZn or other selected proteases (trypsin, papain, acid protease, pepsin or alkaline protease) for comparison, HPLC analysis showed that the hydrolysate peak time of HypZn was approximately 13–20 min, with a time span of 7 min (Fig 7B), and the hydrolysate peak times of the other proteases were approximately 11~25 min, with a time span of 14 min (Fig 7C–7G). Therefore, the hydrolysates (peptides) produced by HypZn were distributed in a narrower length range than those produced by other proteases; thus, the uniformity was improved. In particular, HypZn-hydrolyzed peptide segments contained few small molecule oligopeptides, similar to trypsin-hydrolyzed peptides, according to which there were almost no product peaks after 20 min (Fig 7B and 7C). The hydrolysates of the other proteases, however, contained some oligopeptides (Fig 7D–7G). Reducing small molecule oligopeptides is helpful for reducing the bitterness of hydrolysates because small molecule oligopeptides are the main factor that contributes to the formation of bitterness [39]. Combined with a wide pH range, inactivation at low temperature, tolerance to high concentrations of salt and high enzyme activity, the application of HypZn in the food field is worthy of further research.

## 4 Conclusion

A new metalloproteinase, HypZn, was identified from *A*. *niger*. Protein sequence analysis showed that HypZn belonged to the Metzincin family of astacin-like proteases. The optimal reaction temperature of HypZn was 35°C, which was lower than that of reported metalloproteinases, and it could be inactivated by treatment at 50°C for 30 min. Compared to casein substrates, HypZn exhibited a higher hydrolysis efficiency and activity for soybean protein isolates, and the molecular weight of the hydrolysates was more uniform, resulting in fewer oligopeptides with a potential bitter taste. Under high salt conditions (1000 mM NaCl), HypZn still maintained a normal capacity to hydrolyze soybean protein isolates. Considering the above enzymological characteristics of HypZn, it has good application prospects in the food industry, especially in fermented food processing.

## Supporting information

S1 Raw images (PDF)

S1 Original data (RAR)
