# Supplementary material for: Cloning, expression and characterization of metalloproteinase HypZn from Aspergillus niger
Source: PLoS One. 2021 Nov 11;16(11):e0259809. doi: 10.1371/journal.pone.0259809 (PMC8584677; doi:10.1371/journal.pone.0259809)
Supplement: S1 Raw images — (PDF) [file pone.0259809.s001.pdf]

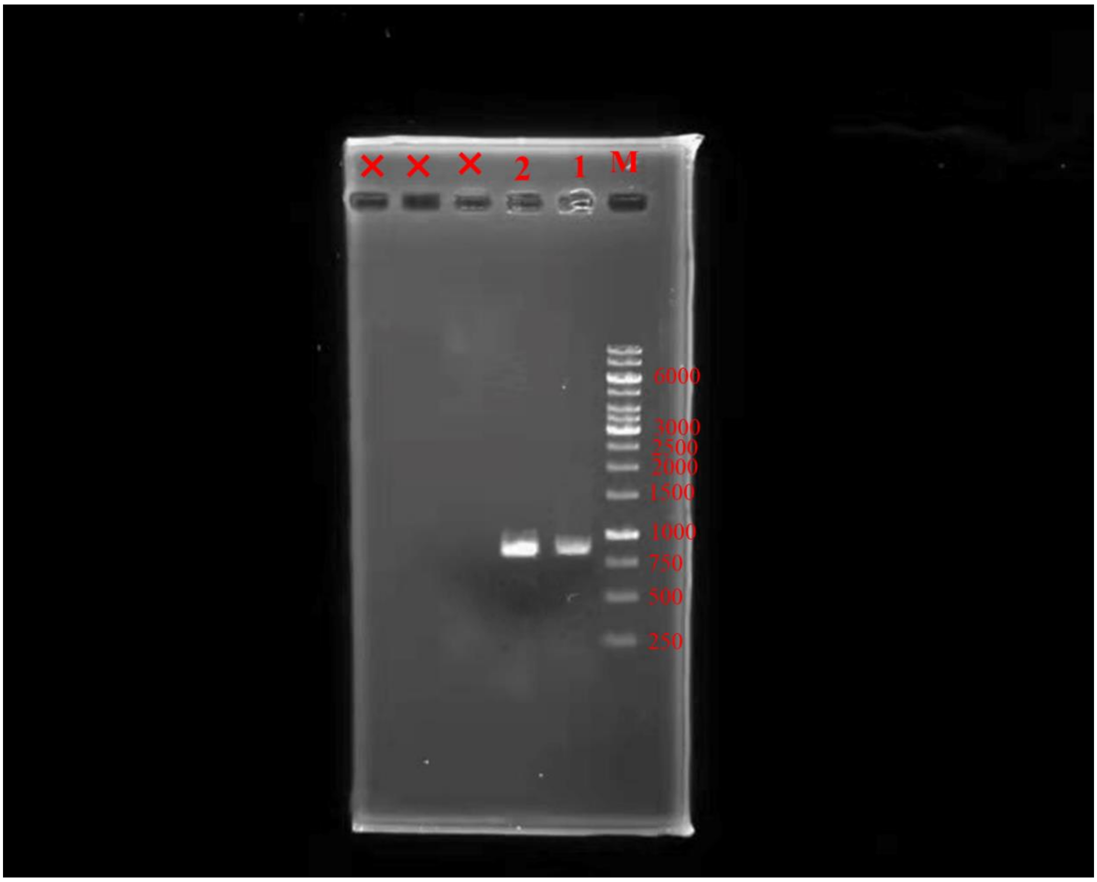

Fig. 2 was generated from this original image. UV transilluminator was used to capture the image.

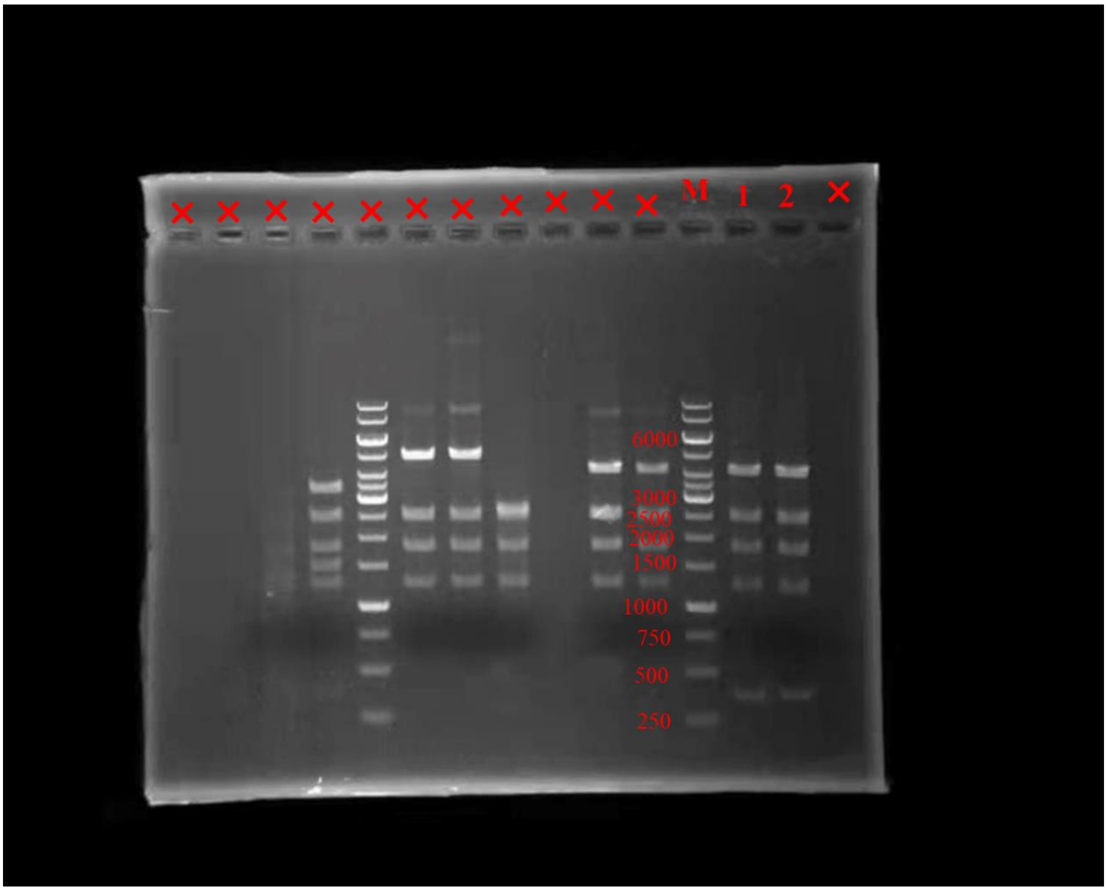

Fig. 3 was generated from this original image. UV transilluminator was used to capture the image.

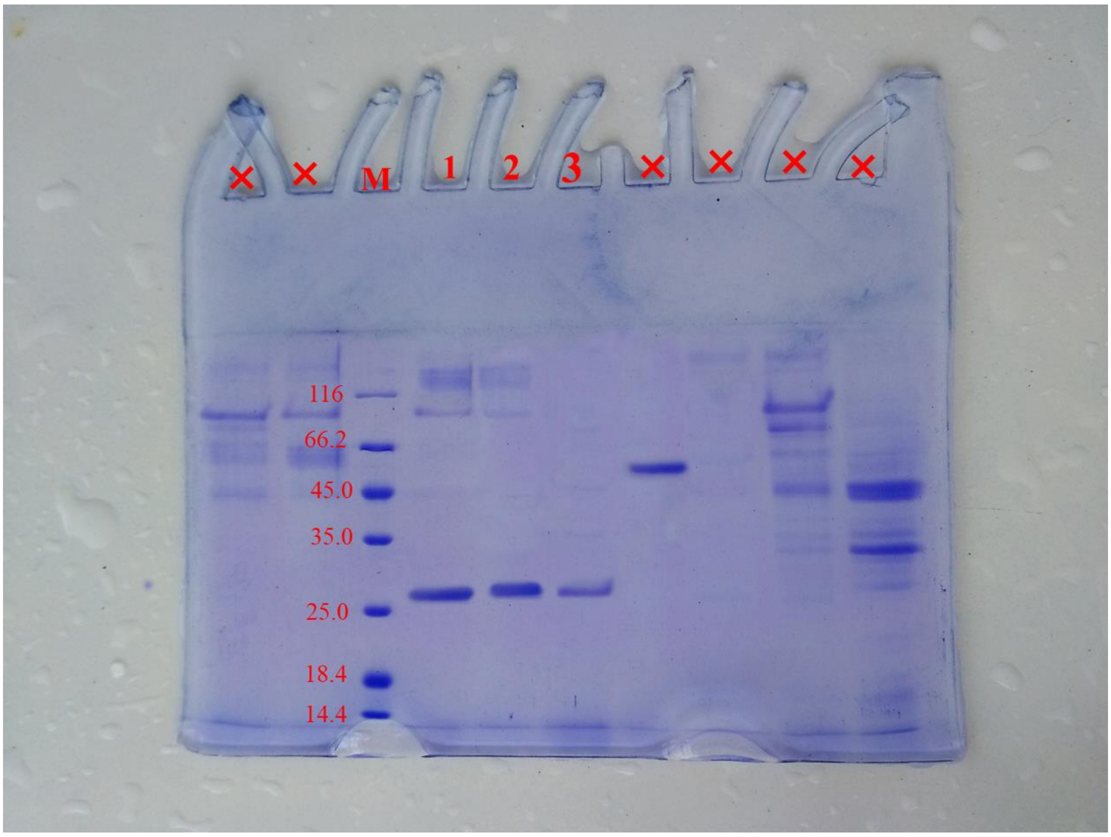

Fig. 4 was generated from this original image. A phone camera was used to capture the image

a

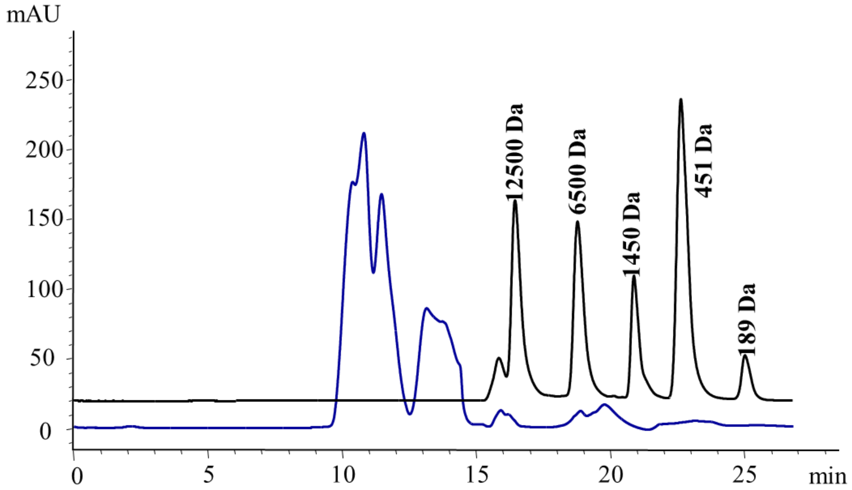

Fig. 7a was generated from this original image. The liquid chromatogram (Agilent 1260) was directly exported to Microsoft word from HPLC chemstation, and then the curve was made bold for observation.

b

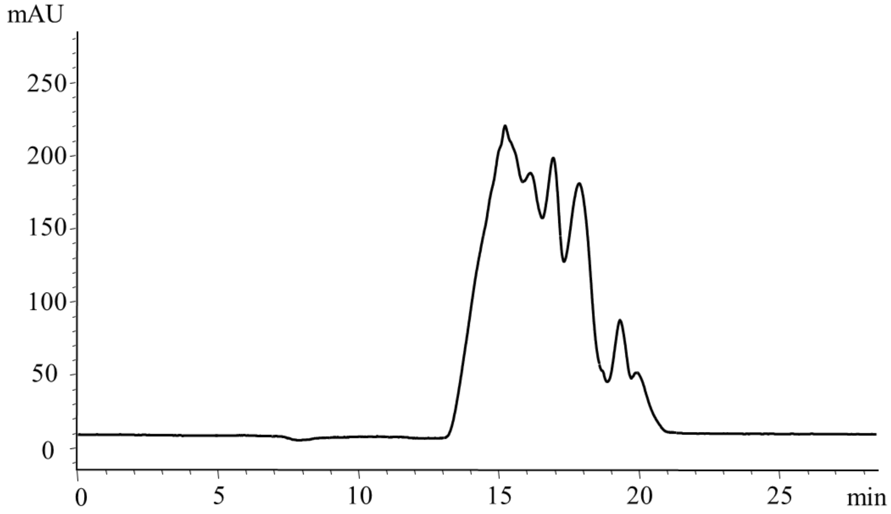

Fig. 7b was generated from this original image. The liquid chromatogram (Agilent 1260) was directly exported to Microsoft word from HPLC chemstation, and then the curve was made bold for observation. **Fig. 7b** showed the peak times of approximately 10~25 min (product peaks).

c

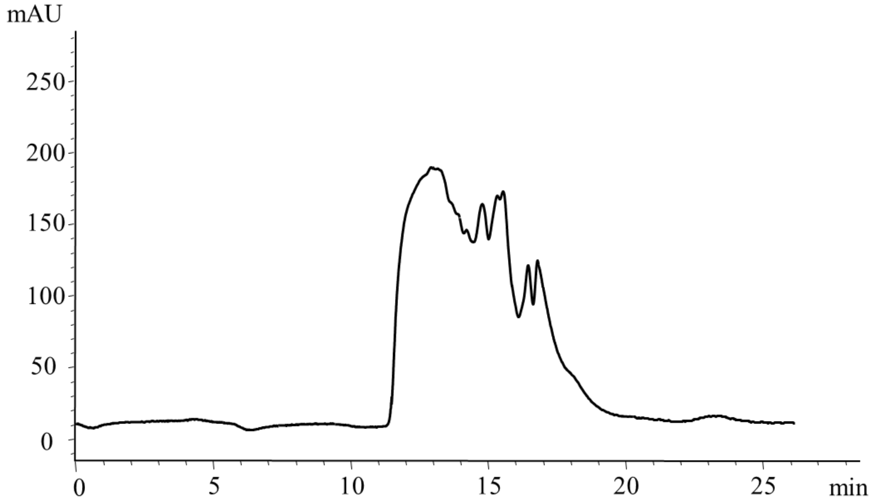

Fig. 7c was generated from this original image. The liquid chromatogram (Agilent 1260) was directly exported to Microsoft word from HPLC chemstation, and then the curve was made bold for observation. **Fig. 7c** showed the peak times of approximately 10~25 min (product peaks).

d

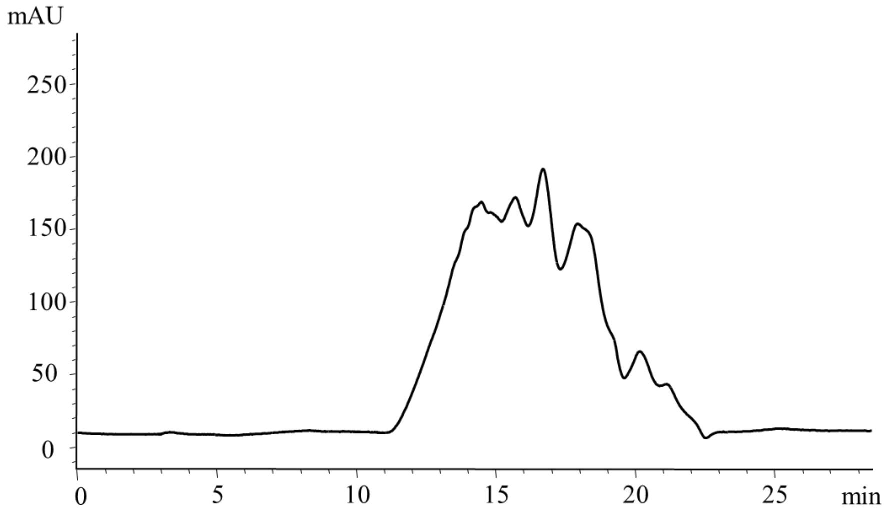

Fig. 7d was generated from this original image. The liquid chromatogram (Agilent 1260) was directly exported to Microsoft word from HPLC chemstation, and then the curve was made bold for observation. **Fig. 7d** showed the peak times of approximately 10~25 min (product peaks).

e

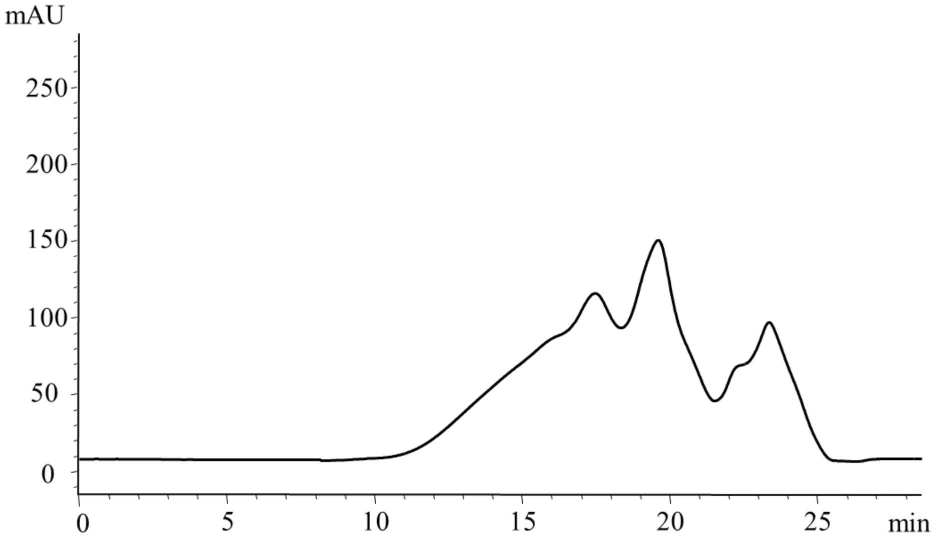

Fig. 7e was generated from this original image. The liquid chromatogram (Agilent 1260) was directly exported to Microsoft word from HPLC chemstation, and then the curve was made bold for observation. **Fig. 7e** showed the peak times of approximately 10~25 min (product peaks).

f

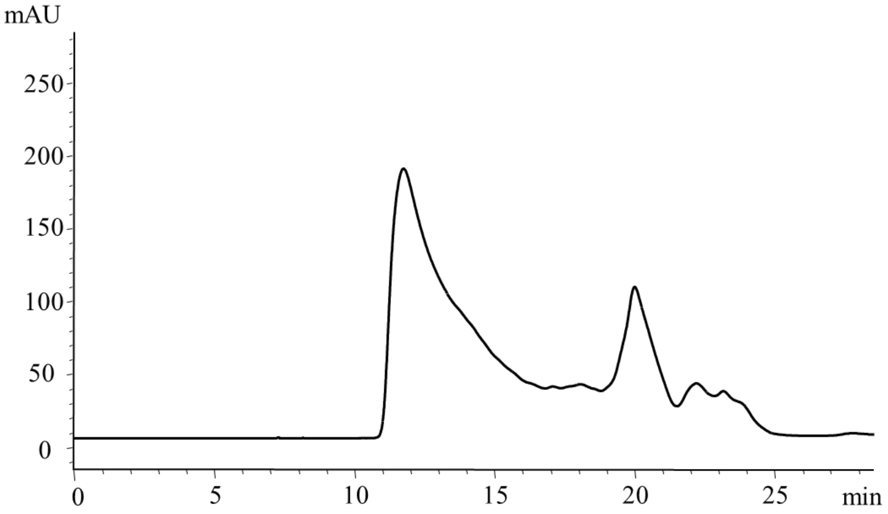

Fig. 7f was generated from this original image. The liquid chromatogram (Agilent 1260) was directly exported to Microsoft word from HPLC chemstation, and then the curve was made bold for observation. **Fig. 7f** showed the peak times of approximately 10~25 min.

g

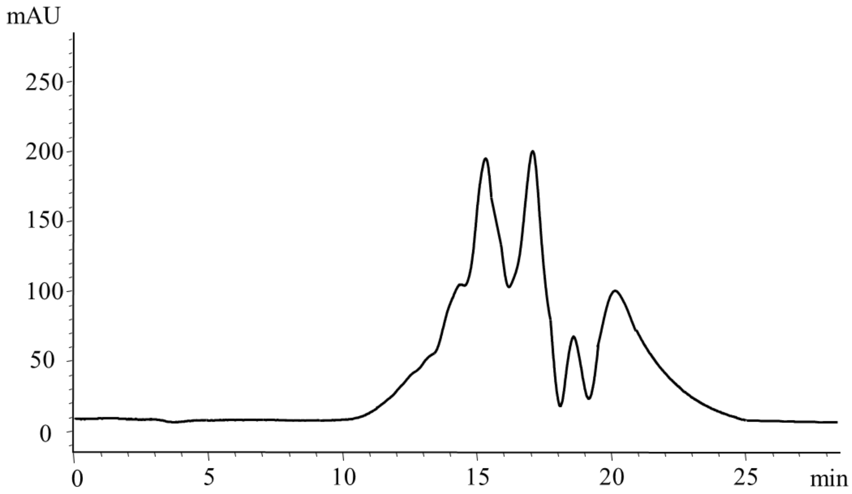

Fig. 7g was generated from this original image. The liquid chromatogram (Agilent 1260) was directly exported to Microsoft word from HPLC chemstation, and then the curve was made bold for observation. **Fig. 7g** showed the peak times of approximately 10~25 min (product peaks).
